# Cell Cycle Progression or Translation Control Is Not Essential for Vesicular Stomatitis Virus Oncolysis of Hepatocellular Carcinoma

**DOI:** 10.1371/journal.pone.0010988

**Published:** 2010-06-07

**Authors:** Sabrina Marozin, Enrico N. De Toni, Antonia Rizzani, Jennifer Altomonte, Alexandra Junger, Günter Schneider, Wolfgang E. Thasler, Nobuyuki Kato, Roland M. Schmid, Oliver Ebert

**Affiliations:** 1 II. Medizinische Klinik und Poliklinik, Klinikum rechts der Isar, Technical University of Munich, Munich, Germany; 2 Medizinische Klinik und Poliklinik II, Klinikum Großhadern, University of Munich, Munich, Germany; 3 Chirurgische Klinik und Poliklinik, Klinikum Großhadern, University of Munich, Munich, Germany; 4 Department of Molecular Biology, Okayama University Graduate School of Medicine, Dentistry, and Pharmaceutical Sciences, Okayama, Japan; The University of Chicago, United States of America

## Abstract

The intrinsic oncolytic specificity of vesicular stomatitis virus (VSV) is currently being exploited to develop alternative therapeutic strategies for hepatocellular carcinoma (HCC). Identifying key regulators in diverse transduction pathways that define VSV oncolysis in cancer cells represents a fundamental prerequisite to engineering more effective oncolytic viral vectors and adjusting combination therapies. After having identified defects in the signalling cascade of type I interferon induction, responsible for attenuated antiviral responses in human HCC cell lines, we have now investigated the role of cell proliferation and translation initiation. Cell cycle progression and translation initiation factors eIF4E and eIF2Bε have been recently identified as key regulators of VSV permissiveness in T-lymphocytes and immortalized mouse embryonic fibroblasts, respectively. Here, we show that in HCC, decrease of cell proliferation by cell cycle inhibitors or siRNA-mediated reduction of G(1) cyclin-dependent kinase activities (CDK4) or cyclin D1 protein expression, do not significantly alter viral growth. Additionally, we demonstrate that translation initiation factors eIF4E and eIF2Bε are negligible in sustaining VSV replication in HCC. Taken together, these results indicate that cellular proliferation and the initiation phase of cellular protein synthesis are not essential for successful VSV oncolysis of HCC. Moreover, our observations indicate the importance of cell-type specificity for VSV oncolysis, an important aspect to be considered in virotherapy applications in the future.

## Introduction

Hepatocellular carcinoma (HCC) accounts for the majority of primary liver cancers in adults [1], [2], [3]. Potentially curative therapies such as liver transplantation and surgical resection can be applied only to a small percentage of patients, and while local-regional treatments (e.g. transarterial embolization, percutaneous ethanol injection, or radiofrequency ablation) may be beneficial for some HCC patients, recurrence is frequent and the long-term survival rate remains poor. Given the limited treatment options and poor prognosis, the use of oncolytic viruses, which have the intrinsic ability to selectively replicate in and kill cancer cells, has found application in the treatment of primary and metastatic liver cancers in preclinical studies and early clinical trials [4], [5], [6].

Vesicular stomatitis virus (VSV), a negative-sense single-stranded RNA Rhabdovirus, which has inherent tumor specificity for replication due to attenuated type I interferon (IFN) responses in most of the tumor cells, is an extremely promising oncolytic agent for cancer treatment [7], [8]. Investigation of the host-cell determinants of permissiveness to VSV infection has become particularly essential for the optimization of viral vectors with enhanced oncolytic properties in HCC, while simultaneously maintaining attenuation in the normal surrounding liver tissue, resulting in a wider therapeutic index. In our previous studies, we have identified impairments in the type I IFN signaling pathway, which contributes to the high sensitivity of HCC to VSV [9]. In addition, cell-cycle progression and eukaryotic translation initiation factors (eIF4E and eIF2Bε) have been reported to determine the susceptibility of T-lymphocytes and immortalized mouse embryonic fibroblasts (MEF) to VSV [10], [11]. The block of cap-dependent translation by rapamycin, a mTOR inhibitor, had no appreciable effect on VSV growth in NIH 3T3 cells, but drastically reduced viral yield in activated T-lymphocytes [10]. Consistent with the fact that cell cycle entry is linked to protein synthesis, G0 to G1 phase transition is crucial to sustain VSV replication in activated T-lymphocytes [10].

Activation of the AKT/mTOR signaling cascade is a common feature in neoplastic transformation and plays a significant role in HCC development and progression [3], [12], [13]. Remarkably, over-expression of eIF4E induces rapid cell proliferation and malignant transformation [14], [15], [16]. At the molecular level, eIF4E over-expression results in the increased translation of c-myc, cyclin D1, and vascular endothelial growth factor, which are involved in cycle progression and tumorigenesis [17], [18], [19]. Recently, eIF4E's oncogenic potential was ascribed to MAP kinase-interacting kinases (MNK)-induced phosphorylation [15], [19]. In addition to its role in regulating eIF4E phosphorylation, the MNK modulate the stability/translation of specific mRNAs and control production of inflammatory mediators, as well as cell proliferation and survival [19], [20], [21], [22]. Impaired translation control by a different mechanism, for example increased levels of the eukaryotic initiation factor eIF2B, is also involved in permissiveness to VSV. The immortalization process of MEFs is associated with a dramatic increase in the levels of eIF2B epsilon subunit [11]. Contrary to primary MEFs, the corresponding immortalized cells support highly productive VSV infection [11].

In this work, we have investigated the function of cell proliferation and functional involvement of AKT/mTOR and MNK/eIF4E pathways in HCC cells and their relevance in VSV oncolysis. We used two human HCC cell lines, HepG2 and Huh-7 and the immortalized non-neoplastic human hepatocyte (PH5CH8) cell line, as a model to investigate the impact of cell cycle progression and translational control on VSV replication. The results presented here show that inhibition of cell proliferation does not affect VSV infection in HCC and immortalized non-neoplastic hepatocytes. Furthermore, we show that eIF4E, as well as eIF2Bε initiation factors are not essential in conferring permissiveness to VSV infection. Taken together, our results demonstrate that the mechanisms underlying VSV oncolysis are cell-type specific and indiscriminate generalization could be misleading in practical applications. Moreover, our findings address the potential use of anti-proliferative agents in combination with VSV for more successful therapeutic outcomes in HCC treatment.

## Materials and Methods

### Cell lines and primary human hepatocytes, viruses

Two human HCC cell lines (HepG2 and Huh-7), a kind gift from Dr. Ulrich Lauer (University Hospital of Tübingen), were maintained in Dulbecco's modified Eagle's medium (DMEM) supplemented with 10% fetal bovine serum (FBS), 1% L-glutamine (200 mM), 1% Penicillin/streptomycin, 1% non-essential amino acids and 1% sodium pyruvate. Immortalized human hepatocytes (PH5CH8) were maintained in DMEM:F12 medium. All cell cultures were regularly tested for mycoplasma contamination.

Primary human hepatocytes (PHH) were derived from patients (negative for HBV, HCV and HIV) that underwent surgical resections of liver tumors according to the guidelines of the charitable state controlled foundation Human Tissue and Cell Research, Regensburg, Germany [23]. PHH were kept in culture with HepatoZYME-SFM medium (GIBCO) containing 1% L-glutamine.

Wild-type VSV (VSV-wt) and the mutant strain VSV-M51R were generated as previously described [5], [24]. Virus stocks were produced on BHK-21 cells and stored at −80°C. Titers were determined by plaque assay on BHK-21 cells as described (12, 13).

### Luciferase assay

Dual-Luciferase Assay system (Promega) was carried out as recommended by the manufacturer. Cells seeded in 24-well plates were co-transfected with the IFN-β promoter-luciferase reporter plasmids. The relative light units were normalized by co-transfection of constitutively active Renilla luciferase and presented as fold-induction respective to the basal expression level. Twenty-four hours later, cells were mock treated or challenged with Poly (I:C), added to the medium or transfected or were infected with wild-type VSV or VSV mutant M51R at an MOI of 1 for 16 hours.

### Western blotting

Whole-cell extracts were run on a 10% SDS-PAGE and transferred to nitrocellulose membranes. Total cell lysates were prepared using lysing buffer (Cell lysing buffer, Cell Signaling Technology Inc., Danvers, MA) containing a protease and phosphatase inhibitor cocktail. Protein concentration in the samples was determined using the BCA protein assay kit (Pierce, Rockville, IL). After blocking for 1 hour with 5% skim milk/TBS-Tween, the membranes were blotted with the following primary antibodies overnight at 4°C: Cyclin D1, CDK4, CDK2, phospho-p70KS6, phospho-eIF4E, eIF4E, eIF2Bε (Cell Signaling); VSV-G (Abcam, Cambridge, UK), Actin (Sigma-Aldrich, St. Louis, MO). After secondary staining with anti-rabbit or anti-mouse peroxidase-conjugated Abs (Jackson ImmunoResearch Laboratories, Inc., West Grove, PA), blots were washed three times with TBS-Tween. Protein bands were visualised on Amersham Hyper-Max film by the ECL chemiluminescence system as recommended by the manufacturer (Amersham, Buckinghamshire, UK).

### Viral growth assays

One-step growth curves of VSV-wt and M51R mutant strains of VSV were performed on immortalized human hepatocytes (PH5CH8), HepG2 and Huh-7 cell lines. Cells (1×10^6^/well) were infected at an MOI of 0.01, 0.1 or 1 according to the experiment. After adsorption for 1 hour, the monolayers were washed three times with PBS, and fresh medium was added. Aliquots of culture media were collected, and fresh media was added immediately at 24 hours post-infection. Viral titers were determined by TCID 50, and each time point represents the average of triplicate experiments. For multicycle growth curves or experiments concerning inhibitor treatments or siRNA transfection, cells were infected at an MOI of 0.1, and viral titers were determined at the indicated time points post infection by TCID50. For interferon protection assays, cells were mock-treated or incubated with 500 IU/ml of Universal type I IFN (PBL Biomedical Laboratories) overnight and subsequently infected with VSV-wt for 24 hours.

### Treatment with inhibitors and siRNA transfection

Cells were seeded at 80–90% confluency in 6-well-plates and serum-starved (DMEM/0.5% FCS) for 36 hours. Serum-free medium was refreshed each 12 hours. Following starvation, the medium was replaced with DMEM/10% FCS containing DMSO or the different inhibitors at the indicated concentration. Cultures were pre-treated for 16 hours and virus infections were carried out in the presence of freshly added inhibitors. Chemicals [CDK4 inhibitor #219476 (250–500 nM); roscovitine (50 µM), AKT IV inhibitor (500 nM), Ly294002 (20 µM), rapamycin (50 nM), aphidicolin (2.5 µg/ml), MNK1 inhibitor (CGP57380)] were purchased from Calbiochem-Merck (Gibbstown, NJ). For siRNA experiments, reverse transfection was performed using DharmaFect4 (Dharmacon, Thermo Scientific). Cells in 96 or 24 well-plates were transfected either without siRNA or with 100 nM of scrambled siRNA or specific siRNA according to the manufacturer instructions. siRNAs were purchased from Dharmacon (Thermo Scientific). 48 or 72 hours post-transfection cultures were infected with VSV-wt at an MOI of 0.1. Titers were determined at the indicated time-points post infection by TCID50. Cell lysates from non-infected duplicates were analysed by Western Blot to assess the efficiency of the RNA silencing.

### MTT assay

HCC cells and non-neoplastic hepatocytes were seeded at the concentration of 5×10^3^ cells per well in 96-well plates. After 24 hours, fresh medium containing the indicated inhibitor was applied to the cells. Following an incubation of 36 hours, MTT assay was performed according to the manufacturer instructions (Vybrant MTT Cell Proliferation Assay Kit, Invitrogen). Cell proliferation was calculated as percentage based on control (mock-treated) cells.

### Flow cytometry

Cell cycle was analyzed by assessing the cell cycle phases using flow cytometry (FACScalibur, using Cell Quest software; Becton Dickinson) according to Nicoletti et al. [25]. HCC cells and non-neoplastic hepatocytes were seeded in 10 cm plates and treated with cycle inhibitors or siRNA as previously described.

## Results

### Immortalized human hepatocytes support VSV replication

**Figure 1:**
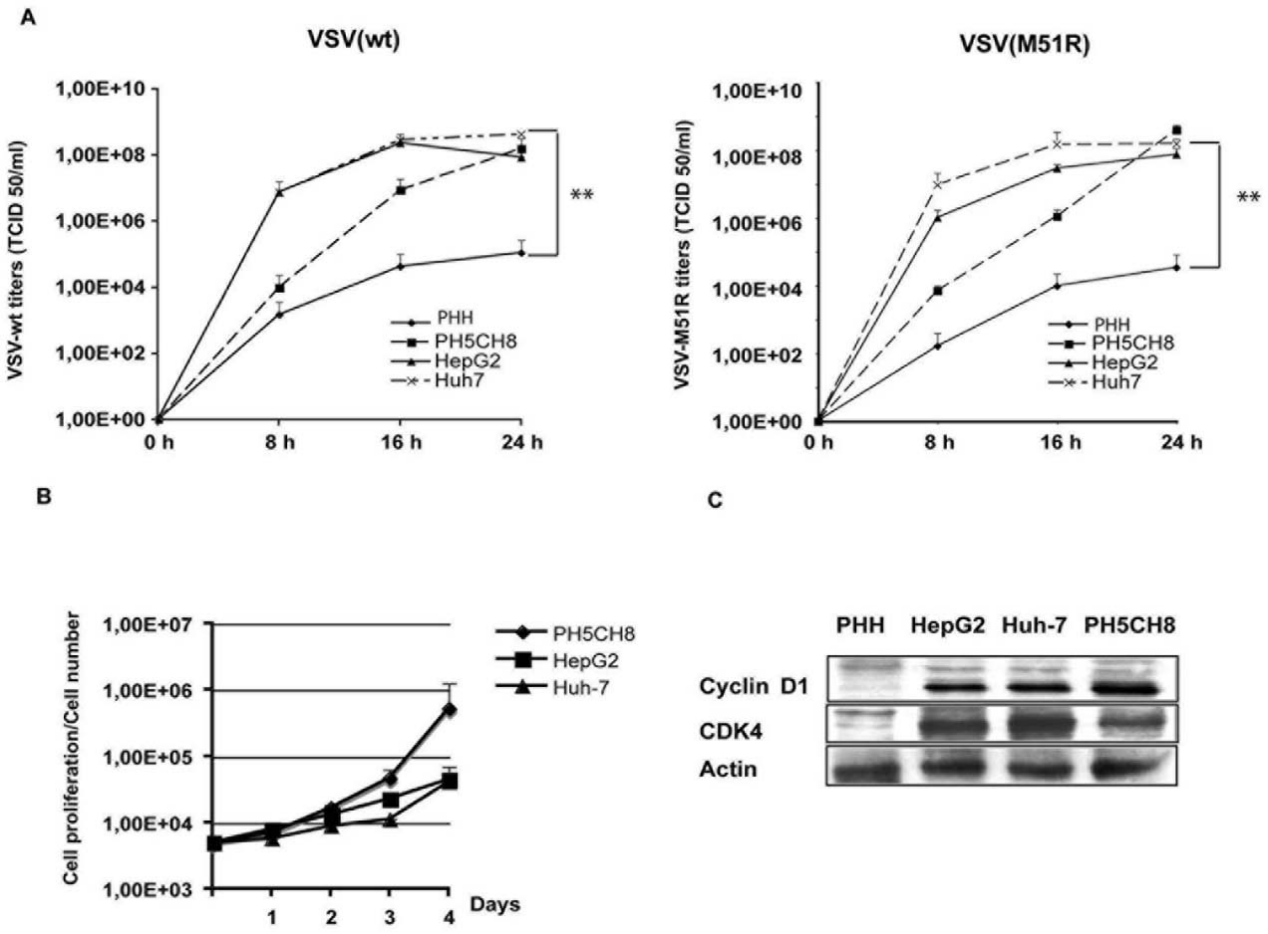
Immortalized human hepatocytes, PH5CH8 cell line. **A)** VSV growth in immortalized non-neoplastic hepatocytes (PH5CH8) was compared to HCC cell lines and primary human hepatocytes (PHH). Cells were infected with VSV-wt and the IFN-inducer mutant VSV-M51R at an MOI of 0.001 and titers were determined at different time-points post-infection as indicated. Data shown are the average of three independent experiments and error bars represent standard deviation. Significance of viral titers in PHH was calculated by comparison with titers in Huh-7 (** p<0.01). **B)** Proliferation of the cell lines: HepG2, Huh-7 and PH5CH8. Cells were plated at the concentration of 5×10^3^ cells per well, and their numbers were determined up to four days after plating by MTT proliferation assay. Data are representative of three independent experiments. **C)** Western blot showing the expression levels of cyclin D1 and CDK4 in HepG2, Huh-7 and PH5CH8 cells compared to PHH.

**Figure 2:**
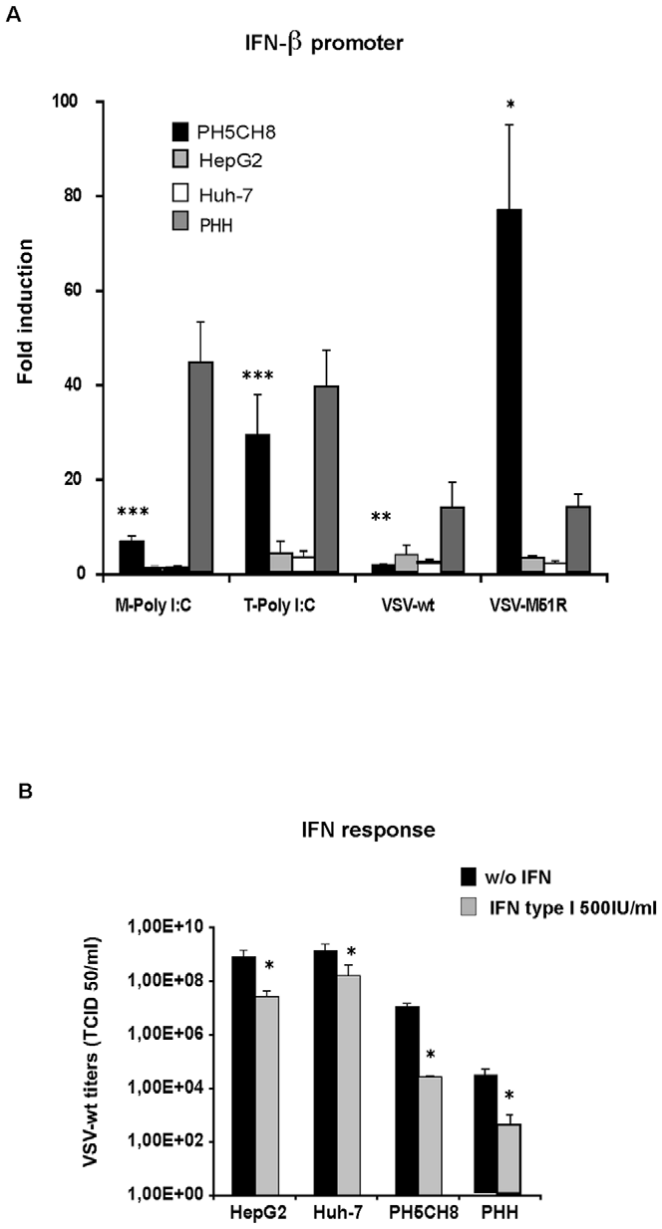
IFN system analysis in PH5CH8 cell line. **A)** Fold induction of IFN-β promoter-Luciferase reporter gene in HCC cell lines (HepG2 and Huh-7), immortalized hepatocytes (PH5CH8) and primary human hepatocytes (PHH). Cells were transfected with the reporter plasmid containing the firefly luciferase gene under the control of the IFN-β promoter. At 24 hours post-transfection, cultures were stimulated by a second round of transfection with Poly (I:C) (T-pIC), Poly (I:C) was added to the medium (M-pIC), or infected with VSV-wt or VSV-M51R. IFN-luciferase activities were measured and normalized to Renilla luciferase (RL) gene used as an internal control. Significance was calculated by comparison with mock-treated cultures expressing basal firefly luciferase activity (* p<0.05; ** p<0.01; ***p<0.001). **B)** Interferon protection assay in PH5CH8 compared to PHH and HepG2 and Huh-7 cells as representatives for HCC. Cells were treated overnight with 500 IU/ml of universal type I interferon (IFN) or simply mock-treated. VSV-wt infection was performed at MOI of 1 and viral titers were obtained 24 hr post-infection. Titers are the mean of at least three independent experiments (* p<0.05).

VSV replicates very efficiently in human HCC cell lines, while it exhibits an attenuated phenotype in primary human hepatocytes (PHH) [9]. We assessed the ability of VSV to grow in immortalized hepatocytes (PH5CH8) in comparison with HCC and PHH (Fig. 1A). Cells were infected with VSV-wt or the mutant VSV-M51R at an MOI of 0.01, and viral titers were determined at different time-points post infection. Recombinant VSV-M51R contains a mutation in its matrix (M) at position 51, which results in an IFN-inducing phenotype [26]. Both VSV strains were able to efficiently replicate in HCC cell lines. Consistent with our previously reported data, the titers in PHH were about 3 logs lower. In PH5CH8 cells, both viruses replicated to a similar extent as in the cancer cell lines analyzed. Although the titers were lower at earlier time-points post-infection (8–16 hours) than those observed in HepG2 and Huh-7, titers at 24 hours post-infection were similar, indicating a more efficient ability of PH5CH8 cells to support viral replication in comparison to non-transformed primary hepatocytes. We have previously shown that resistance to VSV could be partially ascribed to the ability of PHH to mount an efficient innate immune response upon viral infection, while HCC cells have lost the capacity to induce IFN and, therefore, have increased permissiveness to VSV [9]. The ability of PH5CH8 to proliferate in vitro, was assessed by MTT assay and expression of G1-phase cyclins (cyclin D1) and cyclin-dependent kinases (CDK4). Immortalization by the SV40 large T antigen induces active cell proliferation in PH5CH8 to a level comparable to HCC cells (Fig. 1B). Furthermore, expression levels of cyclin D1 and CDK were increased compared to PHH (Fig. 1C). In order to verify the IFN response in PH5CH8 towards VSV, we have performed a dual luciferase assay using an IFN-β promoter (Fig. 2A). Transfection of synthetic dsRNA (Poly I:C) was able to trigger promoter activity in PH5CH8 to levels comparable to those in PHH, as already described by Kato and colleagues [27]. Additionally, in PH5CH8 cells, the IFN promoter was efficiently activated upon infection with the IFN-inducing mutant VSV-M51R. Pre-treatment with IFN induced protection from VSV infection in PH5CH8 and PHH with reduction of viral titers by approximately 2 logs. In contrast, IFN-mediated repression of VSV replication was less efficient in HepG2 and Huh-7 cells (Fig. 2B). Our results indicate that immortalized hepatocytes retain a closer phenotype to PHH in terms of innate immunity.

### Influence of cell cycle on VSV replication

**Figure 3:**
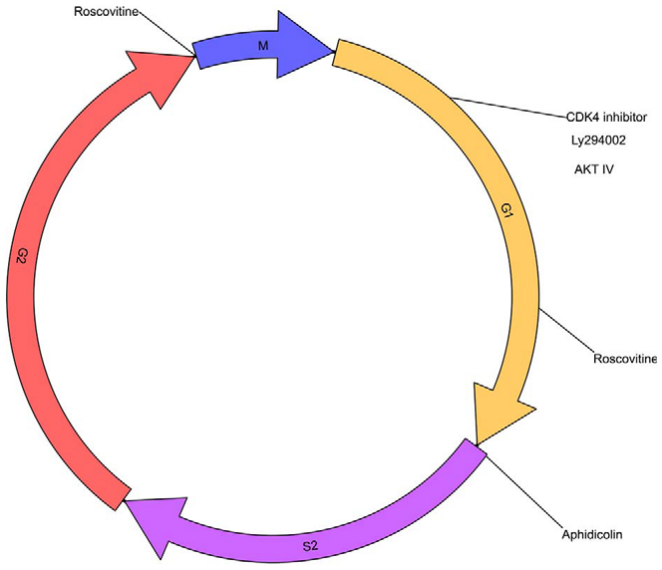
Cell cycle chart and cell cycle inhibitors activity. Scheme of cell cycle inhibitors and their specificity in blocking at a particular phase of cell cycle.

**Figure 4:**
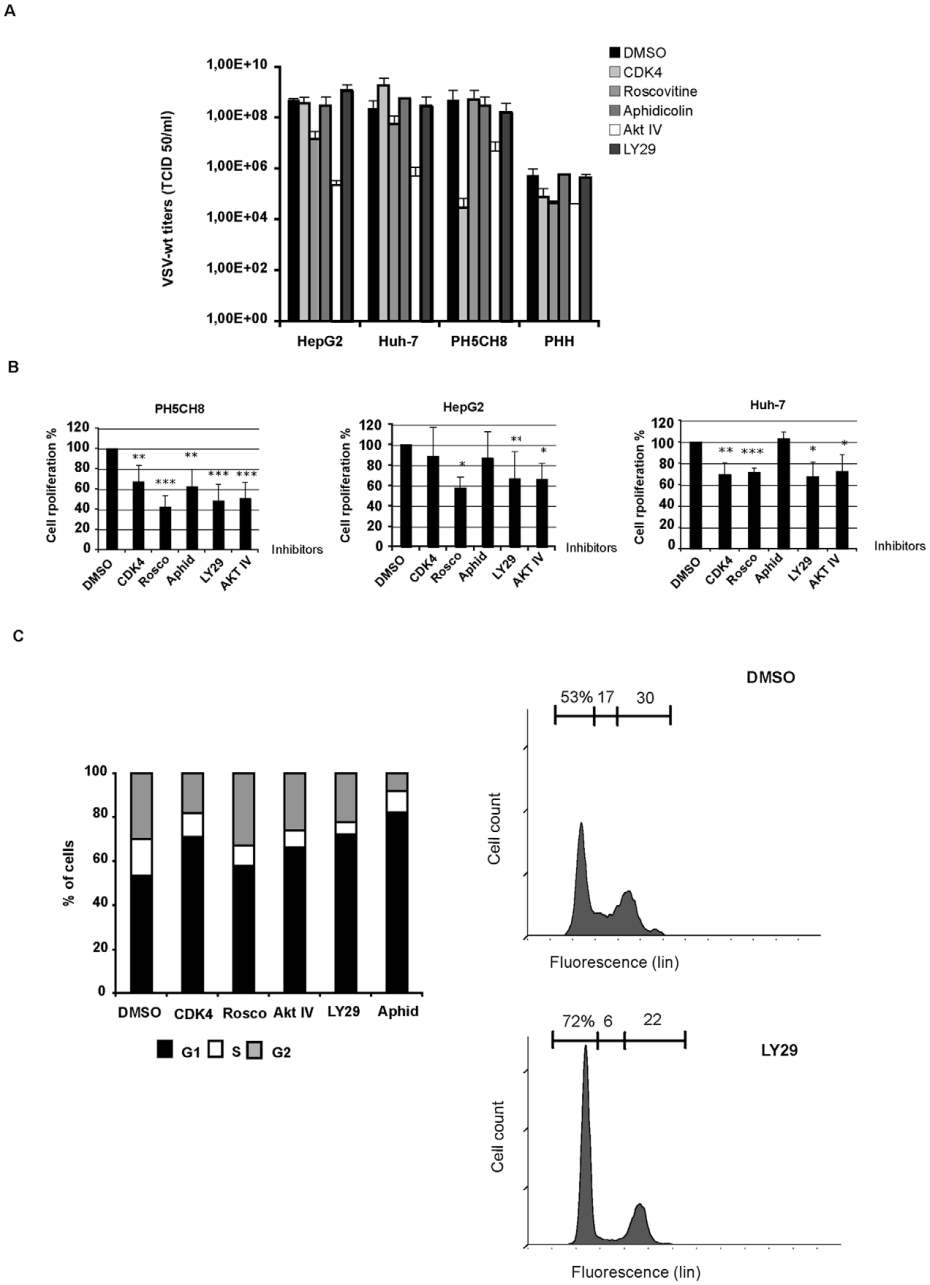
VSV replication and cell-cycle progression. **A)** HCC cells (HepG2 and Huh-7) and immortalized human hepatocytes (PH5CH8) were mock-treated (DMSO) or treated with different cell-cycle inhibitors: CDK4 inhibitor (CDK4); roscovitine (Rosco); Akt inhibitor (AKT IV); Ly294002 (Ly29); Aphidicolin (Aphid). Cultures were infected with VSV-wt at an MOI of 0.1 and viral titers were determined 24 hr post-infection by TCID 50. Data represent the average of 3 independent experiments. **B)** MTT proliferation assay in mock-treated or cell cycle inhibitor-treated cells. **C)** Analysis of cell cycle phases in Huh7 cells after treatment with cycle inhibitors: CDK4 inhibitor (CDK4); roscovitine (Rosco); Akt inhibitor (AKT IV); Ly294002 (Ly29); aphidicolin (Aphid). Samples were prepared in triplicate, and representative data from three independent experiments are shown (p<0.01). Typical FACS pattern of Huh7 cells after treatment with DMSO and LY294002 and PI staining is shown.

Our results so far indicated that the acquisition of proliferating activity by hepatocytes might favour VSV permissiveness. To investigate the contribution of cell cycle and proliferation towards VSV replication, we treated the HCC cell lines, HepG2 and Huh-7, and the immortalized hepatocytes, PH5CH8, with a panel of different chemical compounds that are able to block the cell cycle at different stages (Fig. 3). The CDK inhibitor roscovitine induced only a slight inhibition of viral replication in HepG2 and Huh-7. Early S-phase arrest induced by aphidicolin had no impact on VSV permissiveness in all cell lines tested. A CDK4 inhibitor reduced viral titers only in PH5CH8 cells (Fig. 4A). In addition to specific cell cycle inhibitors, we have also included a PI3K inhibitor (LY294002) and an AKT inhibitor (AKT IV) due to previous reports indicating that AKT is responsible for activation of the viral P protein by phosphorylation [28]. Pre-treated cultures were infected with VSV-wt at an MOI of 1 in the presence of fresh inhibitors for 24 hours. Only the AKT IV inhibitor was able to attenuate viral growth in all the cell lines tested, whereas the PI3K inhibitor revealed no impact on viral replication (Fig. 4A). Efficiency of cell cycle inhibitors was tested by MTT cell proliferation assay. Cell growth in treated cells was evaluated in comparison to mock-treated controls. Significant decrease in proliferation rates was observed in all cell lines after treatment (Fig. 4B). The observation that the impact of cell cycle inhibitors on VSV replication was marginal in our cellular model argues that cell cycle progression is not involved in regulating VSV permissiveness. For all inhibitors, a G1 cell cycle arrest and a corresponding decrease of phase S and G2/M (p<0.01) could be observed by flow cytometry, as shown for the HCC cell line Huh-7 (Fig. 4C). For instance, a G1 cell cycle arrest was readily detectable ranging between 59.6±1.1% (Rosco) to 82±0.1% (Aphid) if compared to DMSO (53±0.5%). After roscovitine treatment, the reduction of the G2/M cell fraction was less prominent if compared to the effect of the administration of other inhibitors. This is in agreement with previous data showing that roscovitine causes a cell cycle arrest in G1 as well as in G2/M (Fig. 4C).

**Figure 5:**
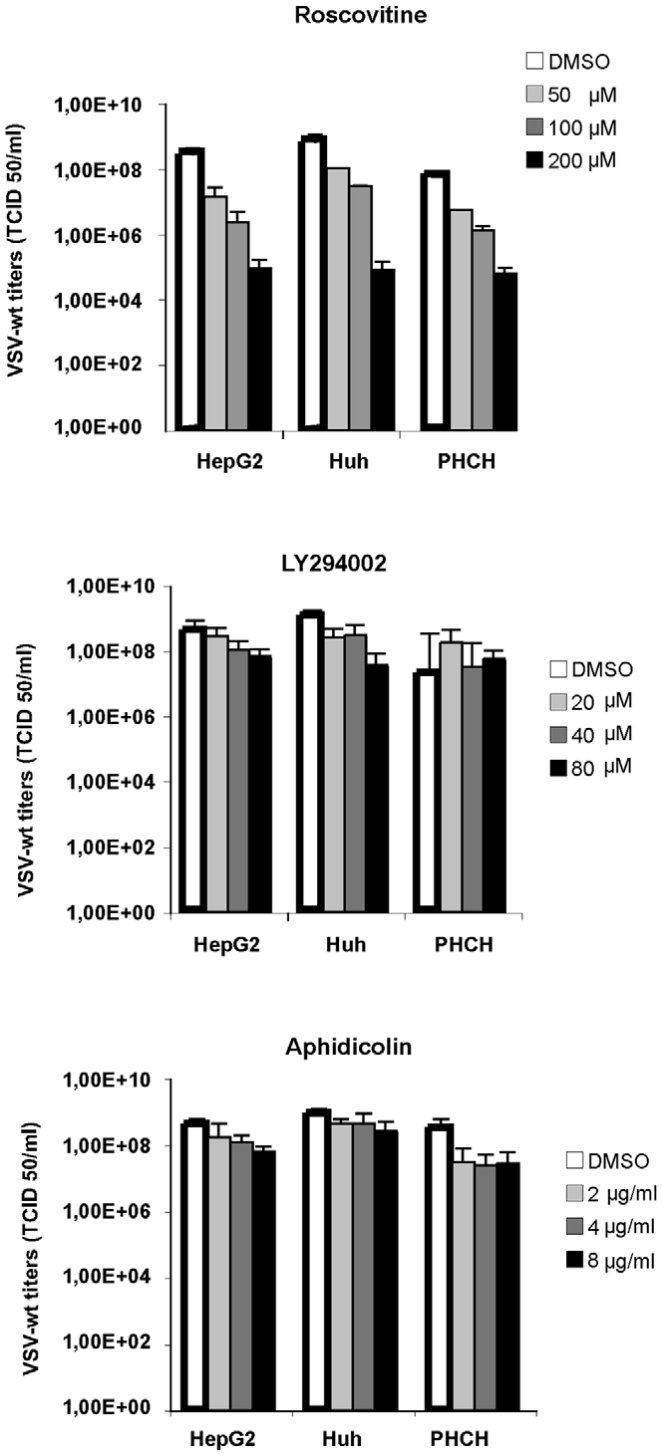
Cell cycle inhibitors. A broader range of concentration was tested until the appearance of cytotoxic effects. HCC cells (HepG2 and Huh-7) and immortalized human hepatocytes (PH5CH8) were mock-treated (DMSO) or treated with increasing concentrations of cell-cycle inhibitors: Roscovitine; Ly294002 and aphidicolin. Cultures were infected with VSV-wt at an MOI of 0.1 and viral titers were determined 24 hr post-infection by TCID 50. Data represent the average of 3 independent experiments.

**Figure 6:**
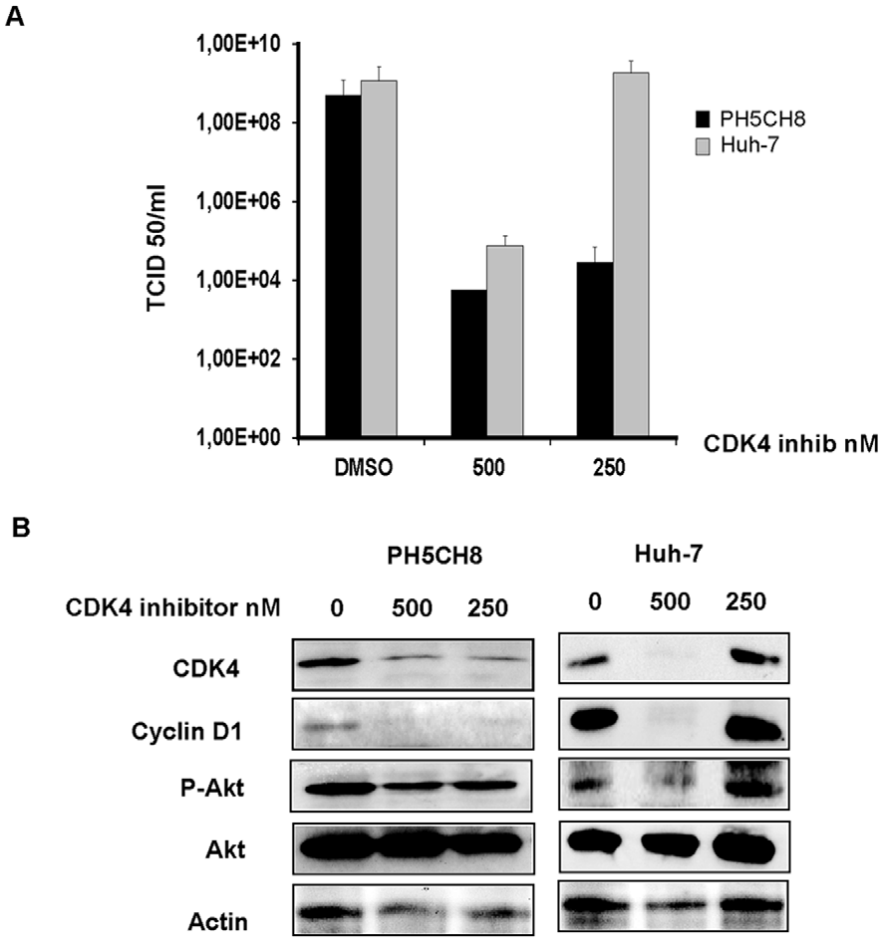
Cell type specificity of the CDK4 inhibitor. **A)** PH5CH8 and Huh-7 cells were treated with DMSO or CDK4 inhibitor at different concentrations. Infection with VSV-wt was performed at an MOI of 0.1 for 24 hr. Viral titers were determined by TCID50. Data represent the average of three independent experiments ± standard deviation. **B)** Protein expression of CDK4, cyclin D1 and Akt in the lysates of the above described experiment was performed by Western blot analysis.

Additionally, we also tested higher concentrations of most of the drugs used to block cell cycle. Ly294002 and aphidicolin did not show cytotoxicity nor viral inhibition even at very high doses. Roscovitine instead decreased viral titers at concentrations 2 to 4 fold higher that the one used to block cell cycle but, as well as AKT IV and CDK4 inhibitors, displayed toxic effects (Fig. 5). The decreased phosphorylation of AKT observed especially in PH5CH8 cells (and in HCCs when applied at higher doses) upon treatment with the CDK4 inhibitor, might explain the effect of the CDK4 inhibitor on VSV replication and, as reported for AKT IV inhibitor, addresses to unspecific activity of this drug (Fig. 6).

**Figure 7:**
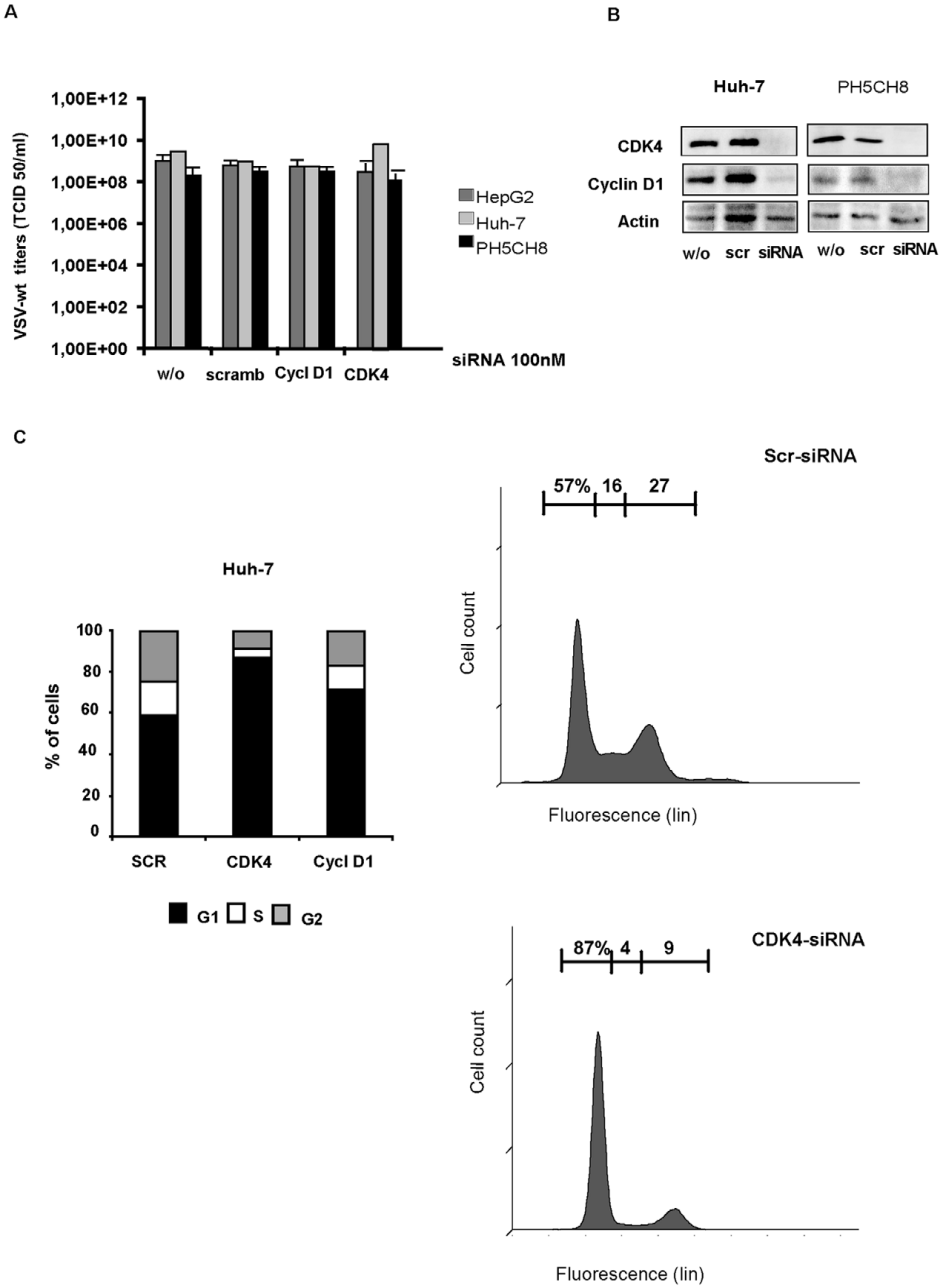
Cell cycle arrest by siRNA. **A)** Cells were transfected without siRNA, with scramble siRNA (scramb) or with siRNA against cyclin D1 or cyclin-kinase (CDK4). Forty-eight hours post-transfection cells were infected with VSV-wt at an MOI of 0.1 for 24 hours. Results show the average of at least three independent experiments. **B)** Mock-infected lysates from PH5CH8 and Huh-7 cells of the experiment describe above are shown for the expression of cyclin D1 and CDK4. **C)** FACS analysis of cell cycle arrest in Huh-7 cells upon treatment with siRNA targeting CDK4 and cyclin D1 or control siRNA (SCR). Experiments were conducted at least three times and triplicate values of one experiment are shown as representative (p<0.001).

To further prove that VSV replication occurs independently of cell cycle, we used RNA interference (Fig. 7A and B). A G1 cell cycle arrest could also be observed after incubation of Huh-7 cells with siRNA targeting CDK4 and cyclin D1 or control siRNA (Fig. 7C). Here, neither the knockdown of cyclin D1 nor CDK4 influences viral titers in HepG2, Huh-7 or PH5CH8 cells reinforcing the argument for cell cycle independent replication of VSV.

### VSV replication is not affected by inhibition of mTOR

**Figure 8:**
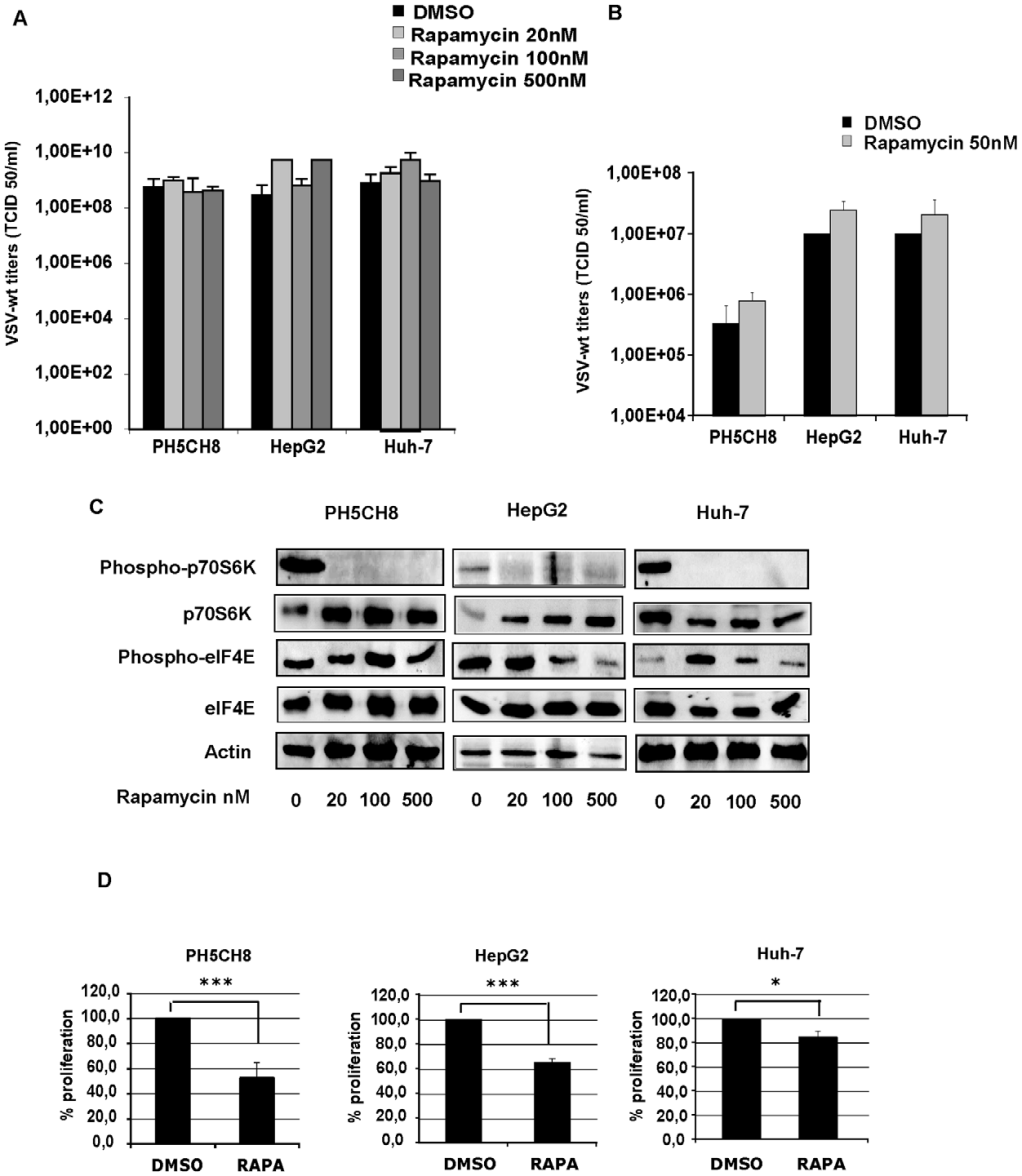
Rapamycin activity on VSV replication. **A)** HepG2, Huh-7 and PH5CH8 cells were incubated overnight with increasing concentrations of rapamycin (20, 100 and 500 nM), or in the case of the controls, DMSO was added. VSV infection was performed at an MOI of 0.1 for 24 hours in the presence of fresh inhibitor. The viral titers shown are the average of three independent experiments. **B)** All cell lines were treated with 50 nM of rapamycin as described above and infected with VSV-wt at an MOI of 0.1. Viral titers were determined at 8 hours post-infection. The data represent at least two independent experiments ± standard deviation. **C)** Cell lysates of mock- and rapamycin-treated cells were analysed by Western blot for detection of the phosphorylated forms of kinase p70S6k and eIF4E and their corresponding base-line expression. **D)** MTT proliferation assay in mock-treated (DMSO) and rapamycin-treated (RAPA) cultures. Data represent the mean of at least three independent experiments ± standard deviation (* p< 0.05; *** p<0.001).

VSV growth has recently been reported to be dependent on cellular translation in T-lymphocytes, as demonstrated by impaired replication in the absence of mTOR and/or eIF4E activation [10]. We therefore analyzed the role of translation initiation in HCC cell lines and immortalized hepatocytes in the support of VSV replication. Rapamycin, the inhibitor of mTOR, was applied at increasing concentrations overnight and cultures were infected with VSV-wt at an MOI of 0.1 for 24 hours in the presence of fresh inhibitor. Rapamycin pre-treatment did not significantly alter viral replication, even when administered at high doses (up to 500 nM) (Fig. 8A). To determine whether rapamycin has an enhancing activity on VSV replication at earlier time points post-infection, we performed the assay with VSV-wt at an MOI of 0.1 for 8 hours in the presence of 50 nM rapamycin. We observed a slight increase in viral titers in rapamycin-treated cells, but differences were not statistically significant (Fig. 8B). The activity of rapamycin was assessed by inhibition of the phosphorylation status of the mTOR substrate S6K (p70 ribosomal protein S6 kinase) and by MTT cell proliferation assay. In all cell lines, rapamycin induced efficient inhibition of mTOR, as demonstrated by the distinct dephosphorylation of the mTOR target S6K (Fig. 8C) and a significant reduction in cell growth (Fig. 8D). When we analyzed eIF4E activation, we observed that rapamycin had only a marginal influence on eIF4E phosphorylation in PH5CH8 and Huh-7 cells, whereas in HepG2 cells de-phosphorylation of eIF4E was observed when higher rapamycin doses were used (Fig. 8C).

### Initiation factors eIF4E and eIF2Bε are not necessary for VSV replication

**Figure 9:**
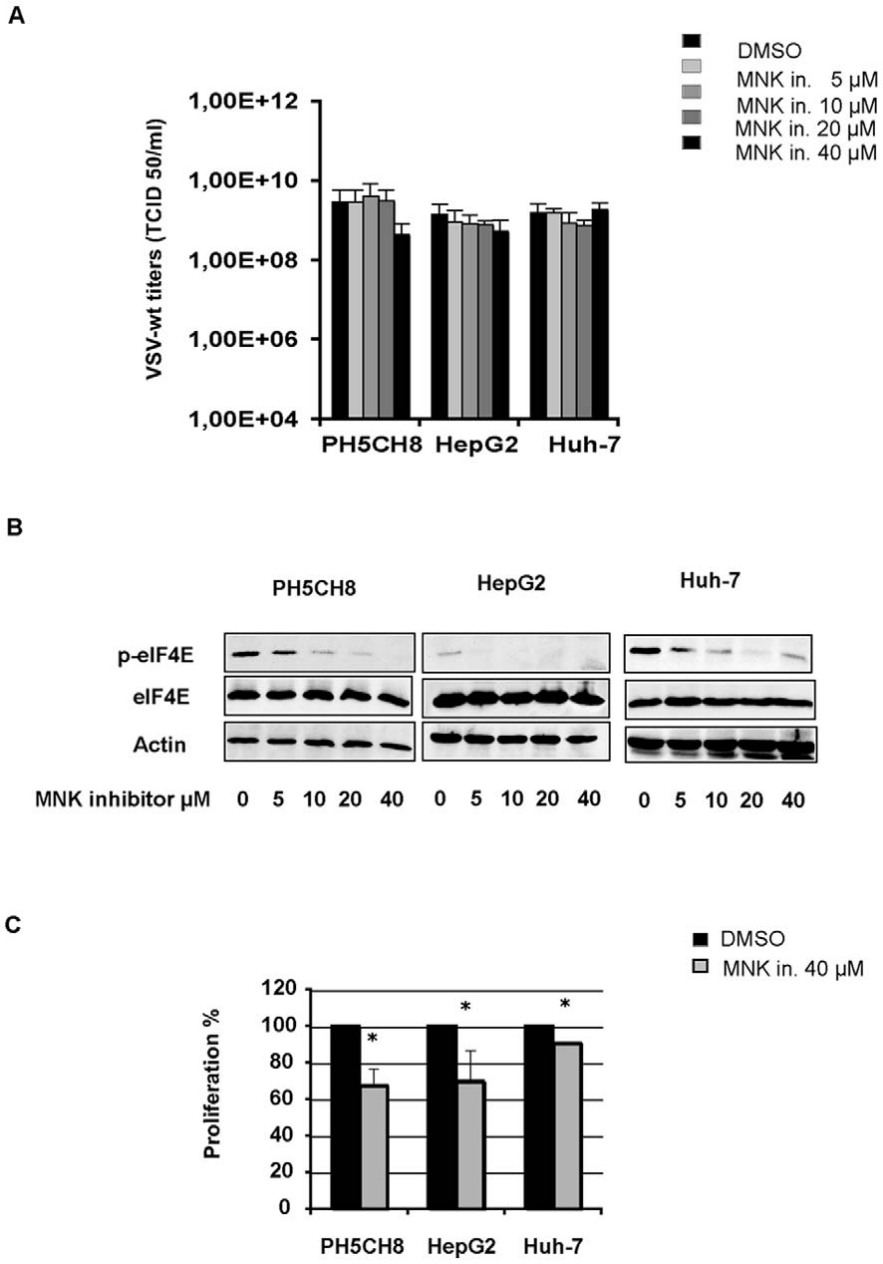
VSV infection does not depend on the phosphorylation status of eIF4E. **A)** Cell cultures, pre-treated with MNK1 inhibitor as described above, were infected with VSV-wt at MOI of 0.1 in the presence of fresh inhibitor. Titers were determined by TCID50 at 24 hours post-infection. Data represent the average of at least three independent experiments ± standard deviation (SD) **B)** Cells were pre-treated with DMSO or the MNK1 inhibitor (CGP57380, Calbiochem) at increasing concentrations for about 36 hours. Phosphorylation of eIF4E was analyzed by Western blot analyses using 100 µg of cell lysates. **C)** MTT assay on HCC and PH5CH8 cell lines treated with 40 µM of MNK1 inhibitor (CGP57380) are shown. Results are the average of three independent experiments ± SD (* p<0.05).

**Figure 10:**
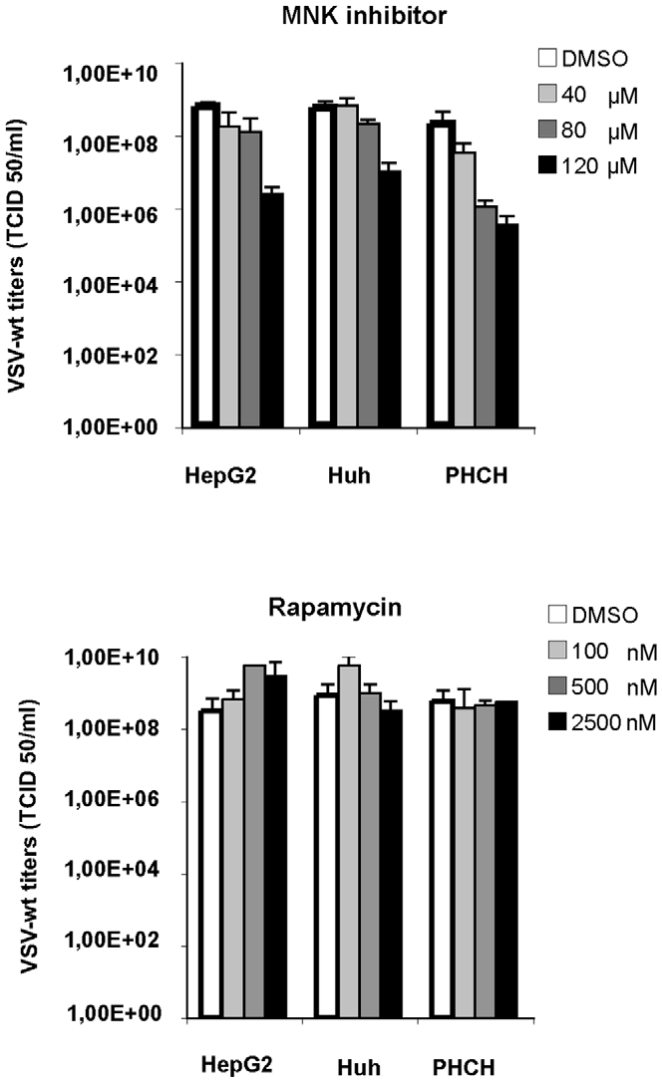
Effects of high doses of MNK inhibitor and rapamycin on VSV replication. HCC cells (HepG2 and Huh-7) and immortalized human hepatocytes (PH5CH8) were mock-treated (DMSO) or treated with increasing concentrations of MNK inhibitor and rapamycin until the appearance of cytotoxicity. Cultures were infected with VSV-wt at an MOI of 0.1 and viral titers were determined 24 hr post-infection by TCID 50. Data represent the average of three independent experiments.

Our aim was to examine whether key players in protein synthesis signaling are important determinants for VSV replication. To determine whether activation of eIF4E contributes to VSV replication, we induced eIF4E de-phosphorylation by the treatment with the MNK1 inhibitor, CGP57380. In fact MNKs, and not mTOR in HCC, are essential for eIF4E phosphorylation [29]. Cells were pre-treated with MNK1 inhibitor for about 16 hours followed by VSV infection at an MOI of 0.1. Infection was performed in the presence of freshly-added inhibitor, and viral titers were determined at 24 hours post-infection. VSV replication was neither affected in non-neoplastic hepatocytes nor in HCC cell lines (Fig. 9A). The phosphorylation state of eIF4E upon MNK1 inhibitor treatment was monitored in uninfected cells by Western Blot analyses (Fig. 8B). Although no changes in viral titers were observed, treatment of uninfected cells with MNK1 inhibitor drastically reduced the amount of the phosphorylated form of eIF4E. This change in eIF4E phosphorylation status was achieved at different concentrations according to the different cell lines (Fig. 9B). MNK1 inhibitor significantly decreased cell growth rates at the highest dose used (40 µM) in all the cell lines tested (Fig. 9C). At higher concentrations of MNK1 inhibitor but not of rapamycin, we observed a reduction in viral titers; however, this reduction is due to an unspecific activity, which occurred in parallel with cyctotoxicity from the high dose of inhibitor applied (Fig. 10).

**Figure 11:**
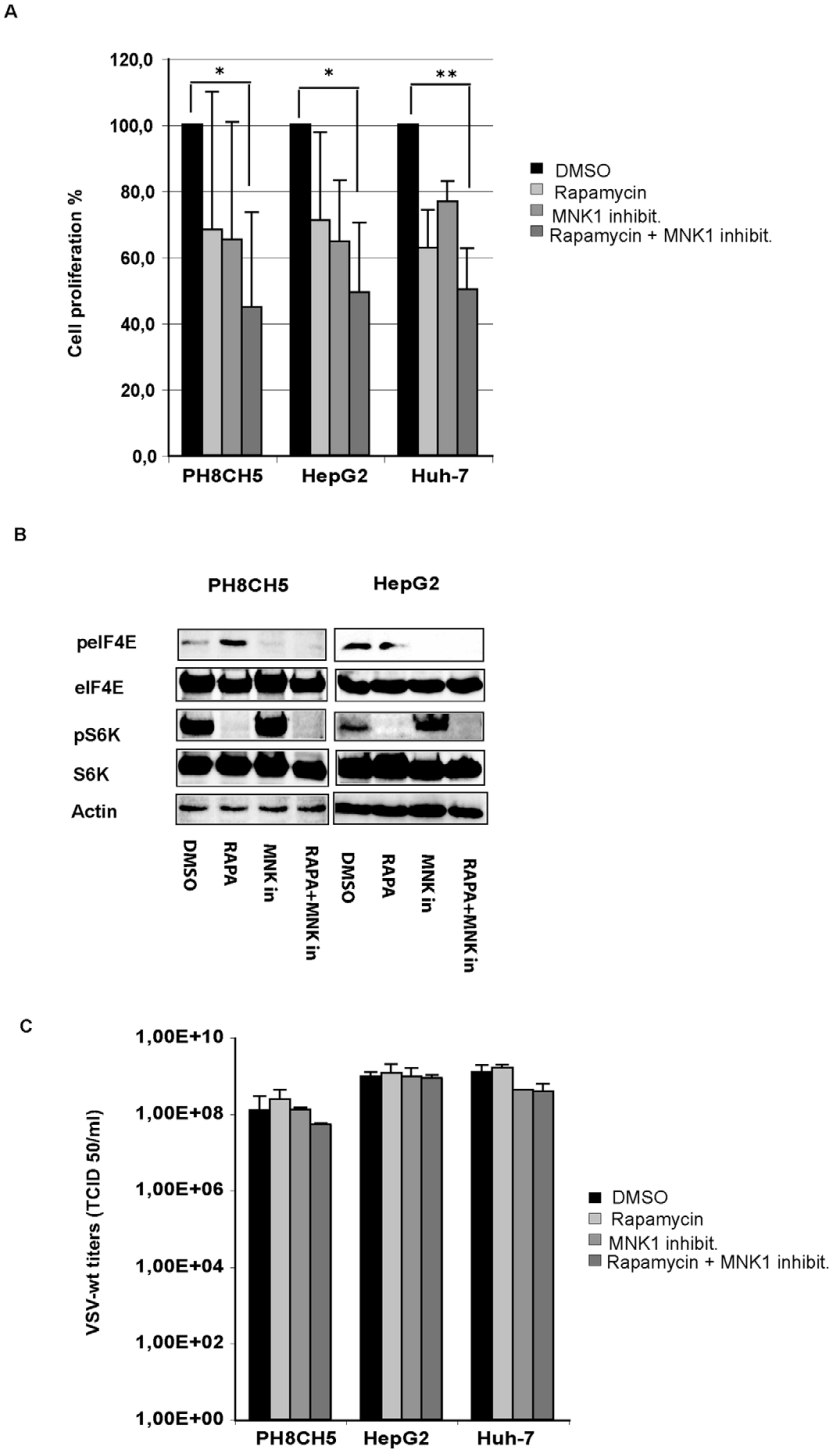
Effects of concomitant inhibition of mTOR and MNK on VSV proliferation. Cells were mock-treated (DMSO) or treated with rapamycin at 50 nm, MNK1 inhibitor at 20 µM, alone or together as indicated. **A)** Cell proliferation assays were performed using the MTT assay. Representative results of at least two independent experiments are shown. **B)** Western blot analysis of lysates obtained by PH5CH8 and HepG2 cell lines mock-treated (DMSO) or treated with rapamycin (RAPA), MNK inhibitor (MNK in) alone or in combination (RAPA+MNK in). The levels of S6K and eIF4E phosphorylated forms were monitored after inhibitor treatment. **C)** Cells were infected with VSV-wt at an MOI of 0.1 for 24 hours. Viral titers represent the mean ± standard deviation of three experiments.

**Figure 12:**
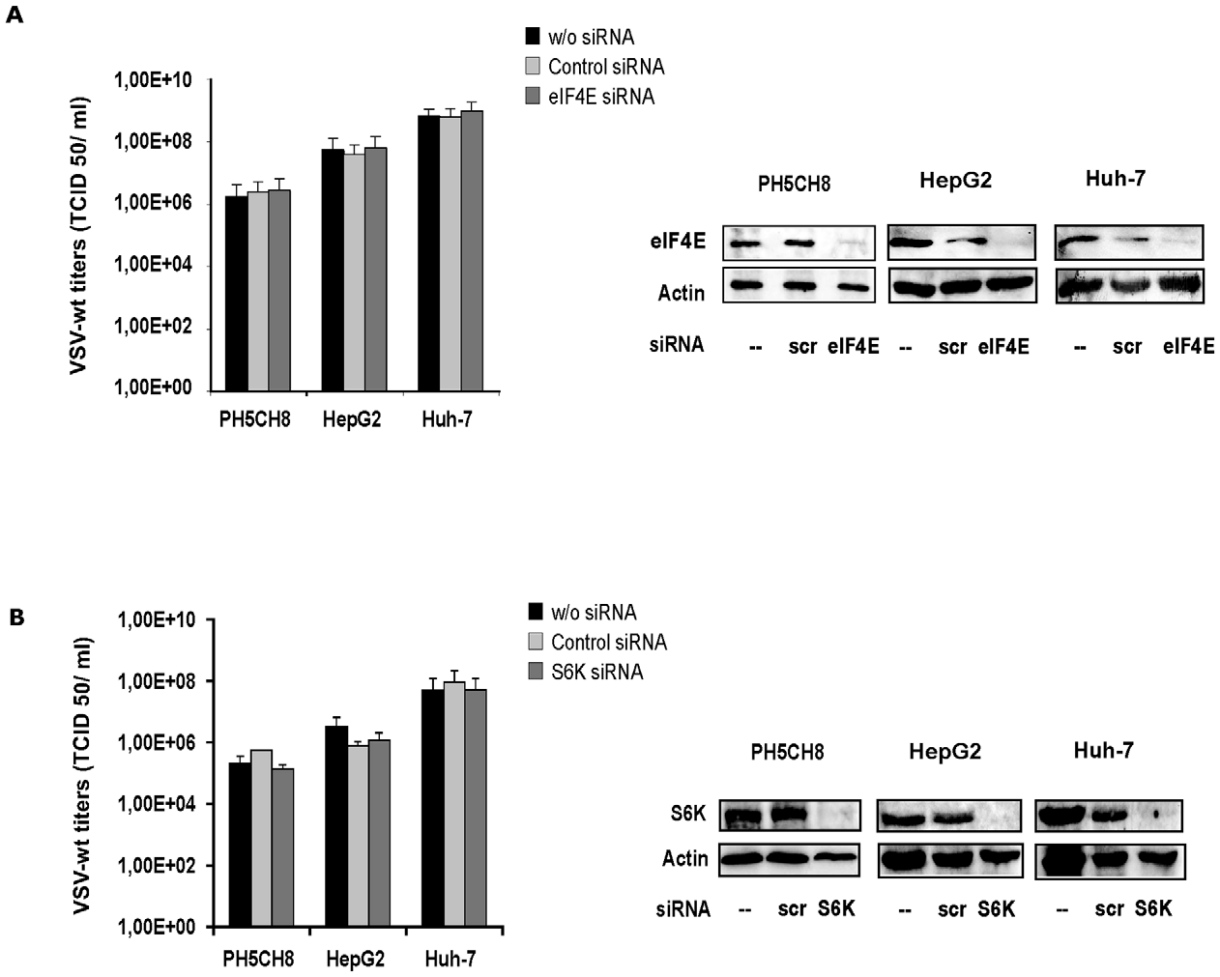
RNA interference assay for eIF4E and S6K. HCC and PH5CH8 cells were transfected with siRNA for eIF4E **A)** or S6K **B)** at a concentration of 100 nM. As controls, cells were transfected in parallel with siRNA scramble or mock-transfected. At 72 hours post-transfection, cells were infected with VSV-wt using an MOI of 0.1 and viral titers were determined 8 hours post-infection. Results are the average of three independent experiments, and error bars indicate the standard deviation. Mock-infected cultures were used to control the efficiency of the mRNA silencing by Western blot analyses. Western blot analysis was performed for each experiment.

Concomitant inhibition of mTOR and MNK1 has been reported to efficiently suppress cell proliferation and protein synthesis in prostate cancer cells [19]. We therefore tested the hypothesis that inhibition of eIF4E phosphorylation might also reinforce the effect of rapamycin on protein translation in our cell model and consequently perturbs permissiveness to VSV infection. First, we measured proliferation of our cell lines in the presence of both inhibitors by MTT proliferation assay. Rapamycin and MNK1 inhibitor only partially decreased proliferation rates when administered alone. The inhibitory effect was more pronounced when the inhibitors were supplied together (Fig. 11A) as described by Bianchini and colleagues [19]. Both inhibitors maintained their specific effects on protein phosphorylation after prolonged incubation (16–24 hours) (Fig. 11B). Moreover, the impact of concomitant inhibition of mTOR and MNK1 on VSV growth was analyzed. Cells pre-treated with rapamycin (50 nM) and MNK1 inhibitor (20 µM), administered alone or in combination, were infected with VSV-wt at an MOI of 0.1 for twenty-four hours. Viral titers obtained in mock-treated cultures were comparable to those in treated cells, with no appreciable differences (Fig. 11C). In order to assess the possibility of phosphorylation-independent involvement of eIF4E, HCC cells and immortalized hepatocytes were treated for 72 hours with siRNA targeting eIF4E and S6K. RNA interference of eIF4E resulted in a drastic decrease of the corresponding protein in all the cell lines tested (Fig. 12A). VSV infection at an MOI of 0.1 was performed in mock-, control siRNA- and eIF4E siRNA-transfected cells and viral titers were determined 8 hours post-infection. Remarkably, VSV replication was not affected by reduced expression of eIF4E (Fig. 12A). Similarly, siRNA knock-down of the mTOR substrate S6K in HCC and immortalized hepatocytes resulted in an efficient knockdown of S6K, while no impact of the S6K depletion on VSV replication was observed (Fig. 12B).

**Figure 13:**
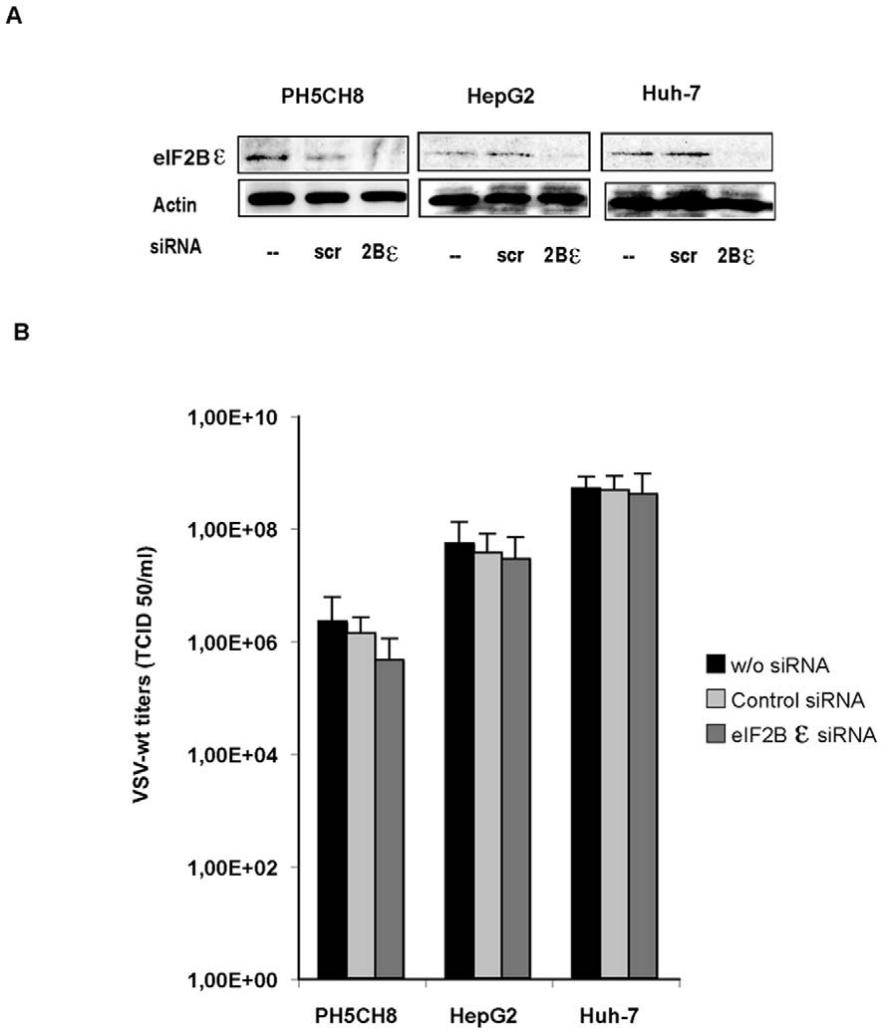
RNA interference assay for eIF2B epsilon (eIF2Bε). **A)** Western blot analysis of mock-infected cultures was performed for each experiment to assess the efficiency of RNA silencing. **B)** HepG2, Huh-7 and PH5CH8 cells were transfected with siRNA for eIF2B**ε** at a concentration of 100 nM. As controls, cells were transfected in parallel with control siRNA or mock-transfected. At 72 hours post-transfection, cells were infected with VSV-wt using an MOI of 0.1 for 8 hours. Results are the average of three independent experiments and error bars indicate the standard deviation.

Increased levels of eIF2Bε are likely to augment permissiveness of transformed MEFs to VSV [11]. Therefore, we tested the role of eIF2Bε subunit in HCC and non-neoplastic hepatocyte cell lines. HepG2, Huh-7 and PH5CH8 cells were transfected with siRNA specifically targeting eIF2Bε and subjected to viral infection with VSV-wt. Knockdown of eIF2Bε expression was monitored by Western blot analyses (Fig. 13A). HCC cells and non-neoplastic hepatocytes, exhibiting a strong reduction in eIF2Bε protein expression did not show a corresponding reduction in viral titers compared to mock- or negative control-transfected cells (Fig. 13B).

## Discussion

Impairments of the type I IFN signaling pathway are thought to be the responsible mechanism contributing to the tumor specificity of VSV replication, explaining its attenuated phenotype in PHH when compared to human HCC cell lines [9]. Although production of IFN plays an important role in host defense, increasing evidence indicates that some additional cellular factors, together with the defective IFN response in cancers cells, might govern the oncolytic specificity of VSV [9]. During malignant transformation, normal growth control mechanisms are partially or completely ablated, thereby inducing abnormal proliferation. While hepatocytes rarely divide in healthy liver tissue, the abrogation of cell cycle checkpoints occurs during tumorigenesis. Recently, it has been shown that VSV infection in primary T-lymphocytes depends on G0/G1 transition and requires translation initiation [10]. For this reason, and because of the notion that a number of different viruses interact with the cell cycle machinery [30], [31], [32], we have investigated the influence of the cell-cycle and cellular protein translation on VSV replication in HCC. In addition to HCC cell lines, we investigated the PH5CH8 cell line derived from human hepatocytes immortalized with the simian virus 40 (SV40) large T antigen [33], [34]. Since PH5CH8 cells, in contrast to HCC, maintain an intact IFN system [27], [35], they represent an ideal cell line for investigating the presence of additional factors contributing to the differential permissiveness to VSV in primary versus non-neoplastic transformed cells. PH5CH8 cells efficiently support growth of VSV-wt and the IFN-inducing mutant, VSV-M51R, but in contrast to HCC cells, they are also able to mount an IFN response upon viral infection with VSV-M51R or stimulation with synthetic dsRNA (Poly I:C), as demonstrated by a reporter gene assay using the IFN-β promoter. PH5CH8 cells are also protected against VSV infection when pre-treated with IFN, indicating a functional IFN signaling pathway. Our results indicate that PH5CH8 cells retain a closer phenotype to primary hepatocytes in terms of innate immunity, but have acquired the ability to proliferate in culture with similar expression levels of G1-phase cyclins and cyclin-dependent kinases as in HCC cells. Based on this observation, we hypothesized that the acquisition of proliferating activity and, therefore, increased protein translation activity, might favor VSV permissiveness, as reported in primary T-lymphocytes [10]. Moreover, a recent publication indicates that disruption of cell cycle in normal rat kidney cells (NRK) is important for VSV ability to kill cells [36]. Chakraborty and colleagues report that VSV infection in NRK cells results in significant cell death during metaphase. To determine whether the same principle applied to our cells, HCC and PH5CH8 cells were treated with a panel of different chemical compounds to block the cell cycle. In addition to specific cell cycle inhibitors, we have also included the PI3K inhibitor, LY294002, and an AKT inhibitor, due to previous reports indicating that AKT activity is necessary for VSV replication [28]. Although we confirmed the inhibitory property of the AKT inhibitor on viral growth, none of the other inhibitors demonstrated an attenuation of VSV replication in the cell lines tested. The CDK4 inhibitor was an exception, attenuating VSV in PH5CH8 cells at the lowest concentration. However, this effect could be due to the influence of the CDK4 inhibitor on AKT activity, as measured by AKT phosphorylation, with PH5CH8 being more sensitive than the other cell lines investigated. In fact, at the lowest dose applied, HCC cell lines were successfully blocked in G1 phase but viral titers were not affected. Interestingly, while the AKT IV inhibitor substantially impaired viral growth, the upstream PI3K inhibitor LY294002 had no effect on VSV growth despite successful de-phosphorylation of AKT (data not shown) and despite inducing G1 phase arrest. We hypothesized that this contradiction was due to the fact that the AKT IV inhibitor is not specific for AKT, but instead has some secondary function. This observation was recently confirmed by Dunn and colleagues [37], who stated that AKT IV is, in fact, a broad-spectrum anti-viral compound with a mechanism differing from its previously reported effect on the PI3K/AKT pathway. These data support our conclusion that the PI3K/AKT pathway is of little relevance to VSV replication.

We then confirmed the independence of VSV growth on cell-cycle phase by using small interfering RNA (siRNA) to specifically target cyclin D1 and cyclin-dependant kinases (CDK) 4. Ablation or reduction of the expression of these cell cycle components did not result in attenuation of VSV replication in any cell line tested but significantly arrested cells in G1. We therefore concluded that, contradictory to what has been seen in primary T-lymphocytes and normal rat kidney cells, cell proliferation is not a key player in supporting VSV replication in HCC. According to Chakraborty and colleagues, VSV preferentially kills cells that undergo mitosis. Accordingly, a block in G1 phase should have reduced cell susceptibility to VSV-mediated cell death in mitosis as hypothesized from the authors in the case of T-lymphocytes. In our study, despite a successful arrest of cell cycle prior to entry into mitosis, we did not observed a reduction in viral titers, based on the general assumption that oncolytic activity is coupled to viral replication. This observation emphasizes once more the importance of cell-type specificity. Kidney cells, as T-lymphocytes, are normal primary cells with a very different molecular make-up from cancer cells, especially concerning their ability to mount an efficient innate immune response, which is irreparably compromised during malignant transformation. Translation rates are particularly robust in cancer cells, and deregulation of the PI3K/AKT/mTOR pathway was reported to contribute to cancer development and maintenance [38]. In some malignant neoplasms, including HCC and derived cell lines, mTOR is highly activated [39] [2], [12]. Arrest of T cells in G1 phase and inhibition of protein synthesis via mTOR and the elongation factor eIF4E activity, inhibits VSV replication [10]. Interestingly however, VSV infection results in reduction of eIF4E phosphorylation, shortly preceding the shut-off of host protein synthesis in tumor cell lines [40].

Our results indicate that rapamycin had no effect on VSV infection in HCC cells and immortalized hepatocytes. Interestingly, rapamycin potently inhibited phosphorylation of S6K kinase, while the phosphorylation status of eIF4E remained almost unaffected. The inability of rapamycin to block activation of eIF4E could explain the efficient replication of VSV in the presence of the inhibitor. However, effective de-phosphorylation of eIF4E by the MNK1 inhibitor, CGP5738, did not lead to attenuation of VSV growth in both HCC cell lines and non-neoplastic hepatocytes, and, furthermore, reduction of eIF4E by RNA interference did not affect virus growth. In an attempt to identify other translational factors involved in VSV permissiveness in HCC, we analyzed the role of the eukaryotic initiation factor 2B (eIF2B). The expression of the ε-subunit of eIF2B is up-regulated in association with increased cell growth and is linked to oncogenesis [41]. Although increased levels of eIF2Bε render transformed MEFs susceptible to VSV [11], knock-down of eIF2Bε mRNA in HCC cells did not alter their permissiveness to viral infection. We speculate that this discrepancy can be ascribed to the fact that the immortalization process in MEFs is associated with a selective block of type I IFN induction [42].

We conclude from our observations that in HCC, enhanced cap-dependent translation and increased proliferation rates are negligible for VSV replication. This finding is in agreement with the observation that VSV selectively induces de-phosphorylation of eIF4E prior to initiation of general translational shut-off, indicating that viral replication does not necessarily rely on active cellular translational pathways [40]. Our results clearly indicate that the mechanisms supporting VSV oncolysis are cell-type specific, and the factors governing VSV permissiveness in T-lymphocytes and normal kidney cells are not applicable to HCC. We can only speculate that these differences can be attributed to substantial differences in viral infection mechanisms in primary cells versus cancer cells. This observation is very important if we consider the possible effects on therapeutic outcomes. Recently, novel antineoplastic agents with strong anti-proliferative effects have been objects of several pre-clinical and clinical studies in treatment of HCC [43], [44], [45]. Based on the results presented here, we can anticipate further studies investigating the combination of anti-proliferative drugs with VSV oncolytic therapy, since no decrease in VSV replication was observed upon inhibition of proliferation or translation.

Mounting evidence suggests that liver cancer stem cells are responsible for HCC initiation. Due to their resistance to chemotherapy and radiation, they are responsible for recurrent tumor formation and account for the failure of conventional therapies [46], [47]. Cancer initiating cells (CIC) have reduced differentiation potential and possess less proliferative capacity. Since we have concluded that active proliferation in HCC is not required for VSV infection, there is the potential that VSV could be effective in eradicating CIC. Although a recent report indicated that VSV failed to successfully infect neuroblastoma or breast cancer CICs [48], it remains to be seen whether or not HCC CICs are permissive to VSV replication. This will be an interesting focus of future experimental investigations.

In conclusion, the application of oncolytic viruses as novel agents in cancer therapy should be based on the understanding of cancer cell biology. Identification of internal cell factors that mediate tumor-specific viral growth is essential for the rational design of viral vectors with potent and selective anti-tumor activity, while minimizing normal tissue toxicity. Combination therapy represents a promising avenue for ongoing translation of oncolytic viruses into clinical practice [49] [50], [51]. In this work, we have provided indications of a potential combination of VSV with anti-proliferative drugs as a rational therapeutic option for HCC.
